# Synthetic anticoagulant heparan sulfate attenuates liver ischemia reperfusion injury

**DOI:** 10.1038/s41598-020-74275-7

**Published:** 2020-10-14

**Authors:** Katelyn Arnold, Yongmei Xu, Yi-En Liao, Brian C. Cooley, Rafal Pawlinski, Jian Liu

**Affiliations:** 1 Division of Chemical Biology and Medicinal Chemistry, Eshelman School of Pharmacy, University of North Carolina, Chapel Hill, NC USA; 2 Department of Pathology and Laboratory Medicine, University of North Carolina, Chapel Hill, NC USA; 3 Division of Hematology/Oncology, Department of Medicine, UNC Blood Research Center, University of North Carolina, Chapel Hill, NC USA

**Keywords:** Biochemistry, Chemical biology

## Abstract

Heparan sulfate (HS) is a sulfated glycosaminoglycan abundant on the cell surface and in the extracellular matrix and has several biological activities including anticoagulation and anti-inflammation. Liver ischemia reperfusion injury is associated with coagulation and inflammatory responses. Here, we synthesized HS oligosaccharides with defined sulfation patterns and show that synthetic anticoagulant HS oligosaccharides limit liver ischemia reperfusion injury in a mouse model. Using a small targeted HS library, we demonstrate that an oligosaccharide that possesses both anticoagulant activity and binding affinity to HMGB1, the inflammatory target, decreases injury greater than oligosaccharides that only bind to HMGB1 or only have anticoagulant activity. HS oligosaccharides may represent a potential new therapeutic option for decreasing liver damage resulting from ischemia reperfusion injury.

## Introduction

Liver ischemia reperfusion (IR) injury is a major complication of surgery during liver transplantation and hepatic tumor resection^1^. Liver surgery often requires the use of the Pringle maneuver to reduce blood loss at the expense of potential IR injury^2^. The initial injury starts with the ischemia phase where blood flow is disrupted to the tissue resulting in a lack of oxygen and flow of nutrients. When blood flow is restored to the tissue, it reestablishes oxygen and nutrients to the ischemic tissue. However, this actually enhances the initial ischemic injury by inducing thromboinflammation which is characterized by disturbances in hemostasis and inflammation^1^. Currently, there are no approved drugs to protect against the liver damage caused by IR injury.

Thrombosis and inflammation are traditionally viewed as separate processes. However, growing evidence supports the relationship between thrombosis and inflammation stimulating and reinforcing one another which is collectively described as thromboinflammation^3^. Thromboinflammation is evident in IR injury^3^, sepsis^4^, and trauma^5^. Damage to the endothelium is central to thromboinflammation pathogenesis. The endothelium acts as an anti-adhesive barrier for the circulatory system by presenting proteoglycans on the cell surface. The heparan sulfate (HS) chains on these proteoglycans can bind to antithrombin III and inhibit coagulation factors FXa and thrombin. The endothelium loses this anti-adhesive and anti-coagulant barrier in thromboinflammatory conditions. Furthermore, tissue factor lies beneath the endothelium and is exposed during vessel wall injury, where it can serve as a potent activator of extrinsic coagulation pathway and subsequent thrombin generation^3^. Additionally, IR injury causes hypoxic cells to release high mobility group box 1 (HMGB1)^6^. HMGB1 has been shown to recruit neutrophils through receptor for advanced glycation end products (RAGE) activation after liver IR^7^. Neutrophil recruitment and infiltration cause further cell death by releasing proteases including myeloperoxidase (MPO)^8^. Severe thromboinflammation can extend beyond the primary affected tissue and lead to multi-organ system failure^3^. Therapeutics that lessen the degree of thromboinflammation are highly desirable to improve patient outcomes^9^.

Here, we show that HS oligosaccharides limit liver IR damage in a mouse model. HS is structurally similar to the anticoagulant drug heparin. HS and heparin are comprised of disaccharide repeating units of glucuronic acid (GlcA) or iduronic acid (IdoA) linked to glucosamine residues that carry sulfo groups^10^. Heparin has higher sulfation and more IdoA residues than HS. Clinical studies using heparin and low molecular weight heparin (LMWH) to treat thromboinflammatory diseases like sepsis and IR are inconclusive on its effect^11,12^. Heparin and its derivatives are complex, structurally uncharacterized oligosaccharide mixtures containing variations in chain length and chemical modifications. The structural heterogeneity makes it difficult to define the relationship of oligosaccharide structure to its biological function. Furthermore, the lack of structurally homogeneous HS oligosaccharides hampers the efforts to exploit the characteristics of HS for use as a therapeutic agent^13^. To address this issue, we have developed a chemoenzymatic method to synthesize structurally specific HS oligosaccharides with high efficiency^14–16^. In this study, we demonstrate that oligosaccharides that possess anticoagulant activity and bind to HMGB1 are more effective in reducing IR-mediated liver injury compared to an oligosaccharide that only binds to HMGB1 or only has anticoagulant activity. By using synthetic HS oligosaccharides, rather than heparin or LMWH, we can distinguish the hepatoprotective effect of oligosaccharides.

## Results

### Liver IR increases liver injury and inflammation

**Figure 1:**
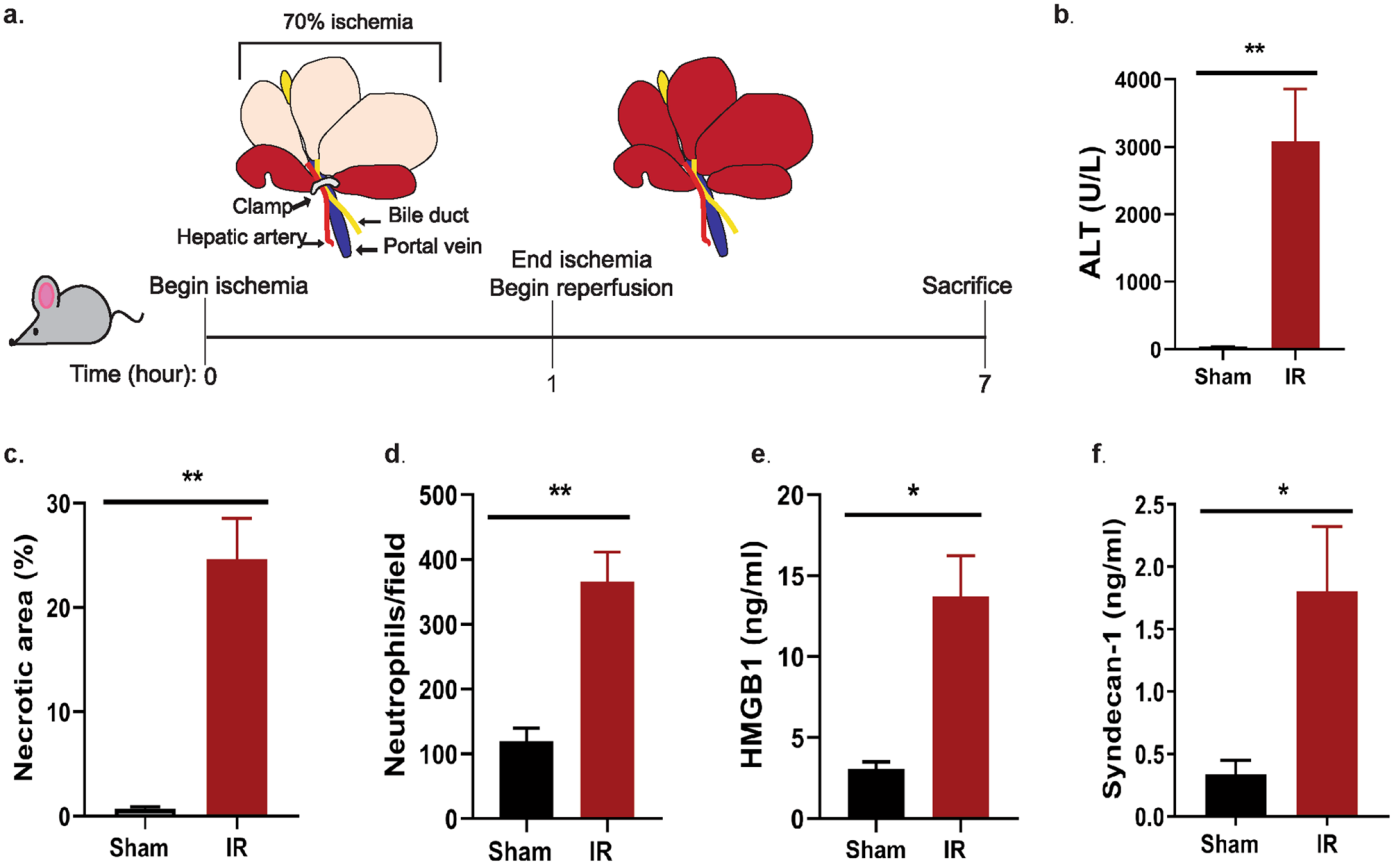
Mouse model of liver IR increases liver injury markers. (**a**) Illustrated timeline of liver IR model. (**b**) Plasma ALT concentration. *P* = 0.0083. (**c**) Percent area of hepatic necrotic area determined by H&E staining and quantified using original magnification of  100x images. *P* = 0.0077. (**d**) Neutrophil infiltration into ischemic lobe quantified by immunohistochemical staining from 200 × magnification images. *P* = 0.0018. (**e**) Plasma HMGB1 concentration. *P* = 0.0129. (f) Plasma syndecan-1 concentration. *P* = 0.0170. Data represent mean ± SEM. Sham n = 4–5, IR n = 4–8. *P < 0.05 and **P < 0.01 by Student t-test.

We used a partial liver IR injury mouse model to evaluate the in vivo efficacy of the oligosaccharides. In this model, a clamp was used to induced ischemia in 70% of the liver (Fig. 1a). After 1 h, the clamp was removed and the reperfusion period begins. Animals were sacrificed after 6 h of reperfusion. Liver injury was measured by elevations in plasma alanine aminotransferase (ALT), necrotic cell area, and neutrophil infiltration into the ischemic liver (Fig. 1b–d). Additionally, IR led to significant increases in plasma HMGB1 (Fig. 1e, P = 0.013) and syndecan-1 (Fig. 1f, P = 0.017). Elevated plasma HMGB1 and syndecan-1 levels are indicators of cell death and endothelium damage^17,18^.

### HMGB1 binds to highly sulfated HS oligosaccharides

**Figure 2:**
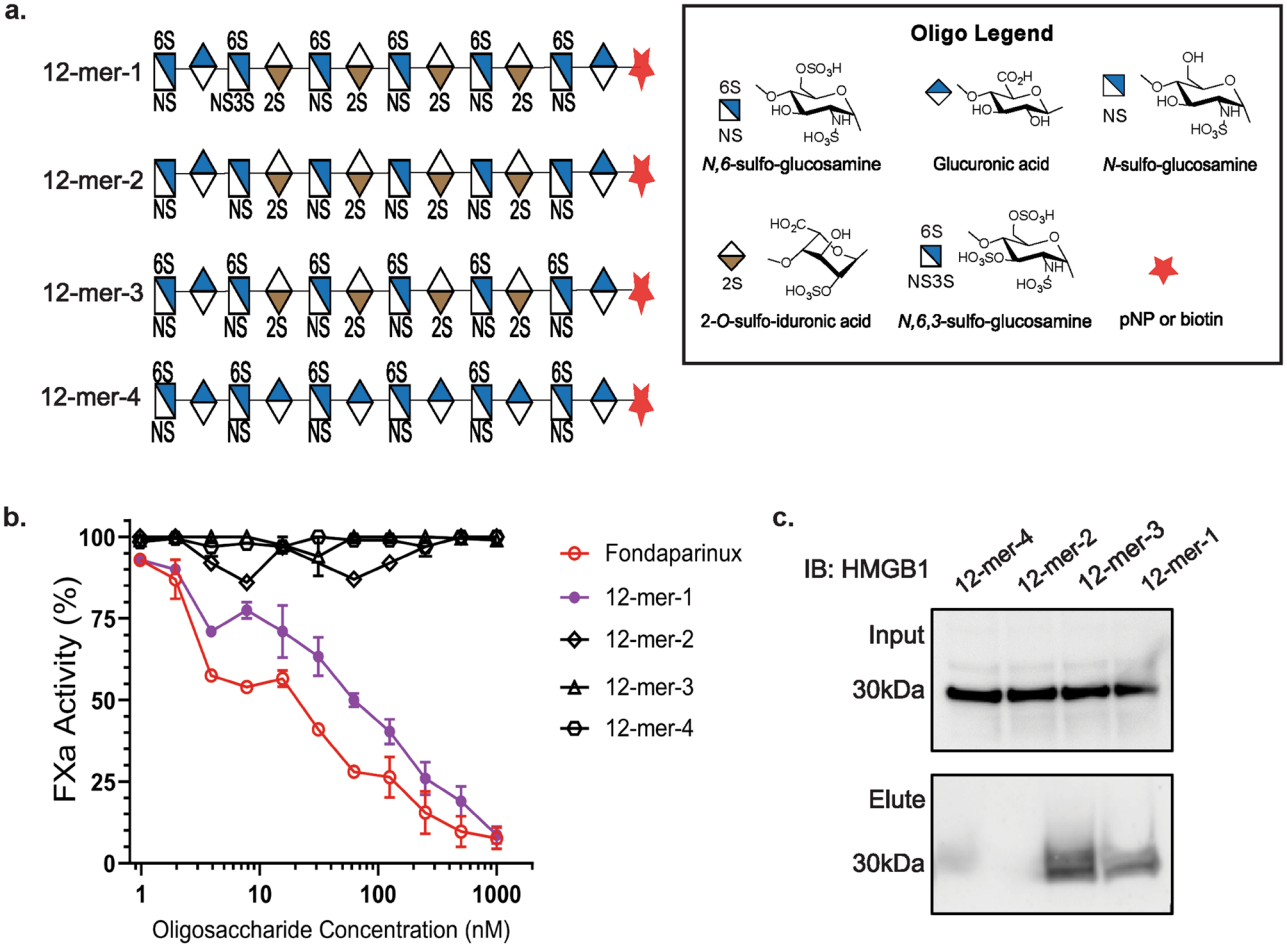
HMGB1 binds to highly sulfated 12-mers. (**a**) Illustration of 12-mer structures prepared by chemoenzymatic synthesis. (**b**) In vitro FXa activity determination for 12-mers with fondaparinux as a positive control. (**c**) Western analysis of HMGB1 pulldown from liver lysate using biotinylated 12-mers.

HMGB1 has been implicated in the damaging inflammation response following liver IR^17,19^. In a recent report, we explored HMGB1 binding to HS oligosaccharides of specific residue-repeat lengths^20^. Here, we tested a panel of 12-mers that vary in sulfation degrees and 2-*O*-sulfo iduronic acid residues. The access to these 12-mers allowed us to further dissect the effect of sulfations or 2-*O*-sulfo iduronic acid residues on HMGB1 binding (Fig. 2a). The panel included four 12-mers covering different sulfation types in the present study. 12-mer-1 has the highest degree of sulfation, carrying 17 sulfo groups and four 2-*O*-sulfo iduronic acid residues. 12-mer-2 has 10 sulfo groups, the lowest among four 12-mers, and contains four *2-O*-sulfo iduronic acid residues. 12-mer-3 contains 16 sulfo groups and four *2-O*-sulfo iduronic acid residues. The structural difference between 12-mer-1 and 12-mer-3 is that 12-mer-1 contains a 3-*O*-sulfo group in one glucosamine residue, but this 3-*O*-sulfation is not present in 12-mer-3. 12-mer-4 contains 12 sulfo groups and has no 2-*O*-sulfo iduronic acid residues. The anticoagulant activity of 12-mer-1 was similar to fondaparinux, a FDA approved anticoagulant drug, as measured by inhibiting the activity of factor Xa (anti-FXa). 12-mer-1 and fondaparinux have anti-FXa IC_50_ values of 63 and 18 nM respectively, while 12-mer-2, -3, and -4 did not display anti-FXa activity and thereby have no anticoagulant activity (Fig. 2b). Next, oligosaccharides appended with biotin tags were used to pull down endogenous HMGB1 from liver lysate (Fig. 2c). Interestingly, 12-mer-1 and 12-mer-3 successfully pull down HMGB1, suggesting that at this size of oligosaccharide, degree of sulfation is an important factor for HMGB1 binding.

### 12-mer-1 decreases liver injury after IR

**Figure 3:**
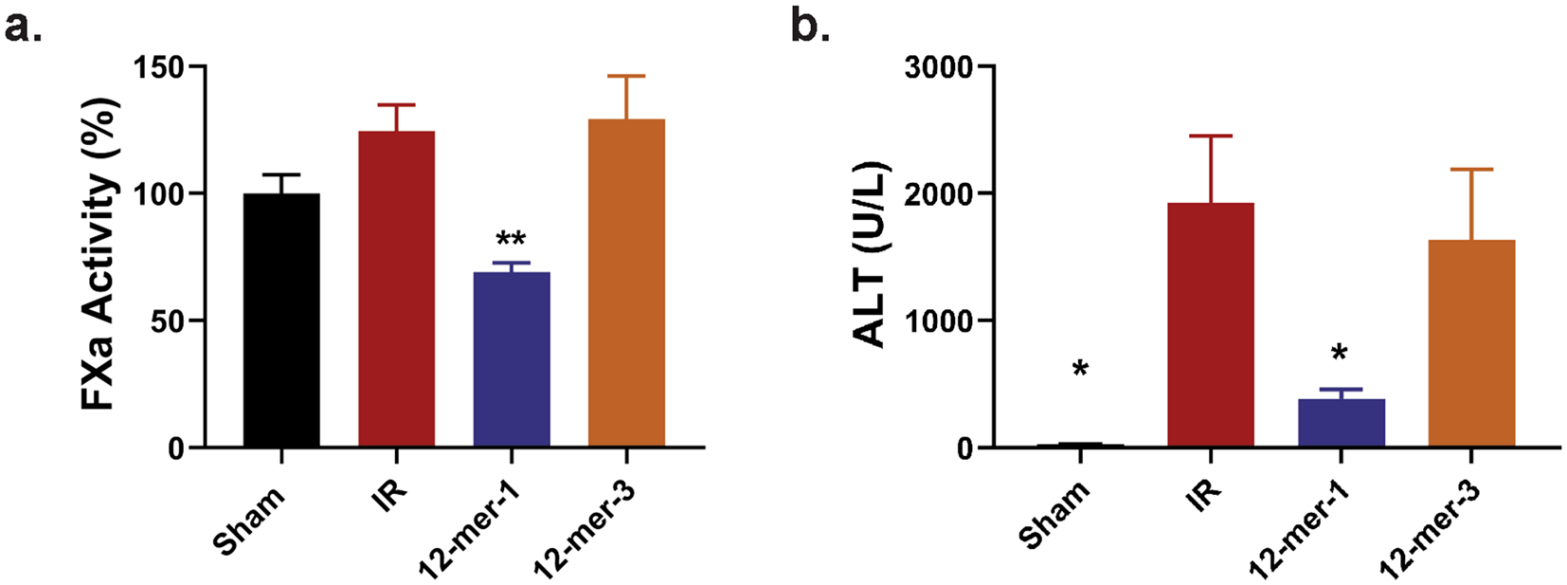
In vivo determination of anticoagulation and liver injury after IR using 12-mer-1 and 12-mer-3. Plasma FXa activity (**a**) and ALT (**b**) was measured from mice that underwent a sham or IR procedure with 12-mer-1 or 12-mer-3 administration. 12-mer-1 significantly decreased FXa activity (12-mer-1 vs IR, *P* = 0.0057) and ALT compared to IR (Sham vs IR, *P* = 0.0233; 12-mer-1 vs. IR, *P* = 0.0480; 12-mer-3 vs IR, *P* = 0.6200). Data represent mean ± SEM. N = 5–6 for all groups. *P < 0.05 and **P < 0.01 by one way ANOVA followed by Dunnett’s test.

**Figure 4:**
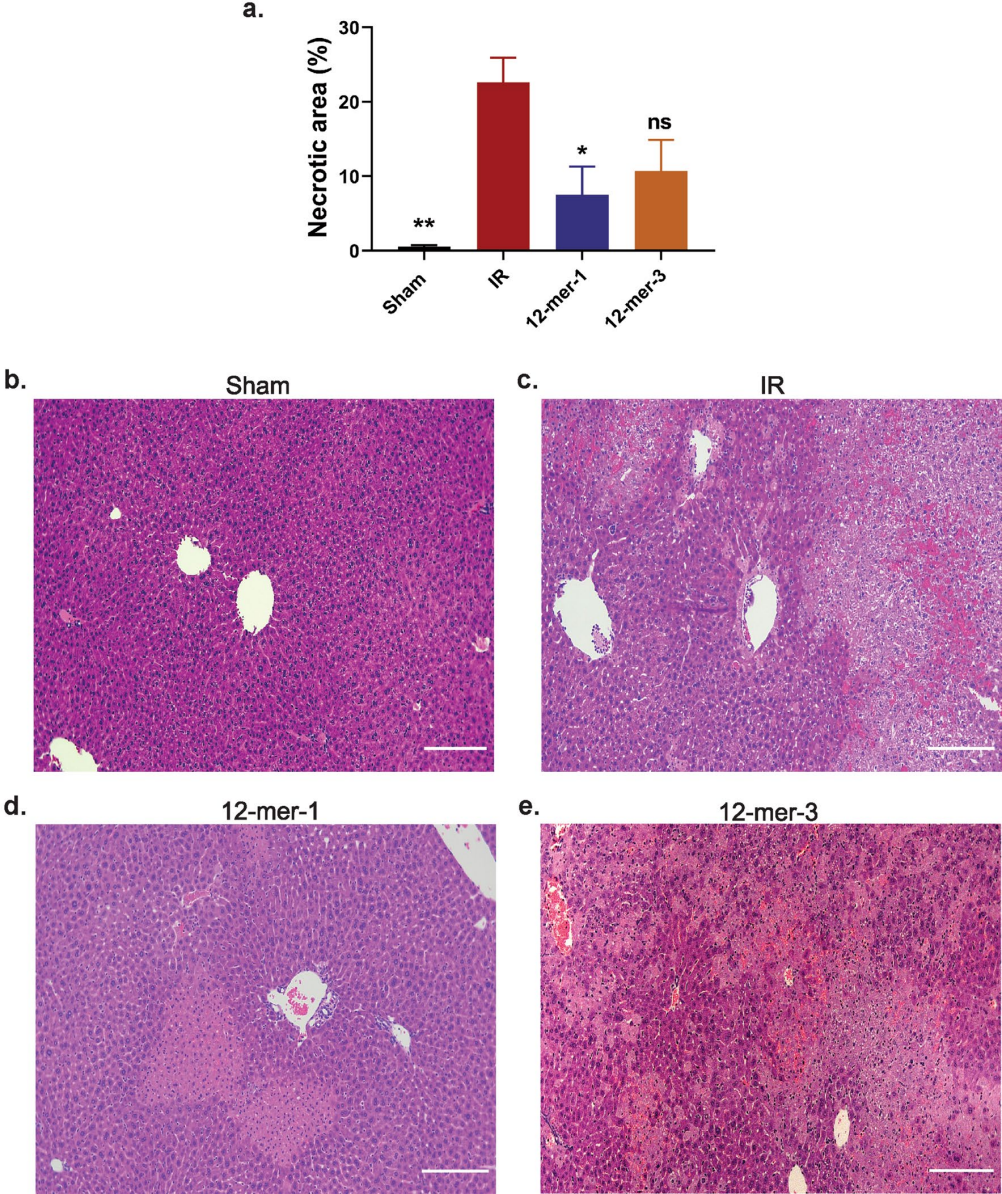
12-mer-1 decreases hepatic necrotic area in ischemia lobe after liver IR. (**a**) Percent area of hepatic necrotic area determined by H&E staining and quantified using original magnification of × 100 images. Sham vs IR, *P* = 0.0078; 12-mer-1 vs. IR, *P* = 0.0287; 12-mer-3 vs IR, *P* = 0.1059. Data represent mean ± SEM. Sham n = 3, IR n = 4, 12-mer-1 n = 6, and 12-mer-3 n = 5. *P < 0.05 and **P < 0.01 vs. IR by one way ANOVA followed by Dunnett’s test. (**b**–**e**) Representative images of H&E stained liver tissue. × 100 magnification. 200 μm scale bar.

Based on the ability to bind to HMGB1, 12-mer-1 and 12-mer-3 were used in the in vivo model of liver IR. The compounds were administered 30 min prior to ischemia. Anticoagulant activity of 12-mer-1 was confirmed in the plasma (Fig. 3a). Although both 12-mer-1 and 12-mer-3 bind to HMGB1, only 12-mer-1 significantly decreased plasma ALT (Fig. 3b; 12-mer-1 vs IR *P* = 0.048, 12-mer-3 vs IR *P* = 0.620). 12-mer-1 also decreased hepatic necrosis in the ischemic liver lobe compared to the IR group (Fig. 4; 12-mer-1 vs IR *P* = 0.0287, 12-mer-3 vs IR *P* = 0.1059). This suggests that 12-mer-1’s anticoagulant and anti-inflammatory properties offer protection against liver IR injury.

### 12-mer-1 decreases neutrophil accumulation and MPO activity of the ischemic liver

**Figure 5:**
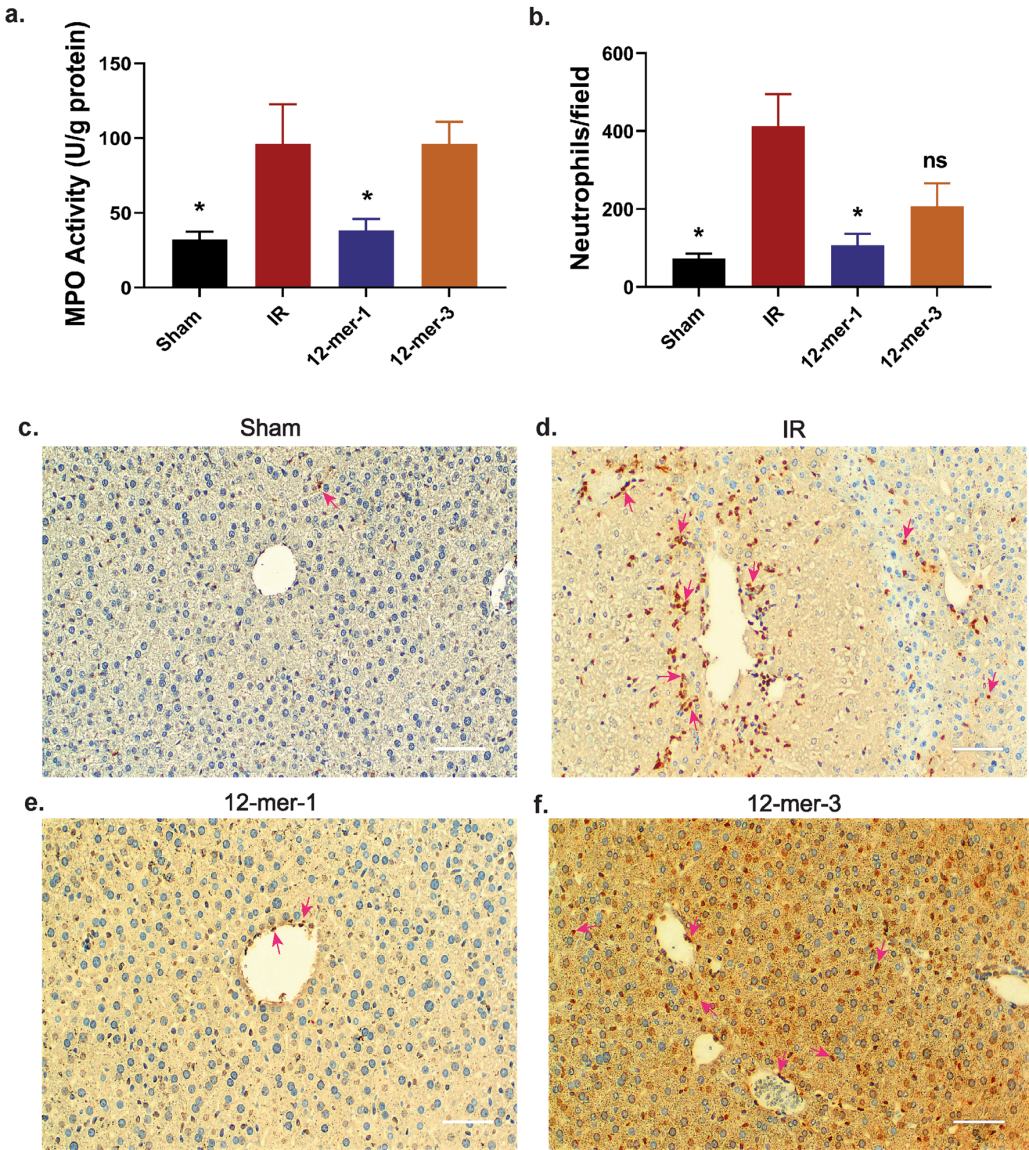
12-mer-1 decreases neutrophil accumulation in the ischemia liver. (**a**) MPO activity measured in sham or ischemic liver lysate. Sham vs IR, *P* = 0.0121; 12-mer-1 vs IR, *P* = 0.0229. (**b**) Quantitation of average neutrophils per 100 × field of view. Sham vs IR, *P* = 0.0248; 12-mer-1 vs IR, *P* = 0.0142; 12-mer-3 vs IR, *P* = 0.0705. Data represent mean ± SEM. Sham n = 6, IR n = 3–4, 12-mer-1 n = 4–6, 12-mer-3 n = 6. *P < 0.05 by one way ANOVA followed by Dunnett’s test. (**c**–**f**) Representative images of neutrophil immunohistochemically stained liver tissue.  200x magnification. 200 μm scale bar. Pink arrows indicate stained neutrophils.

Neutrophil-derived proteases including elastase, MMP-9, cathepsin G, proteinase-3, and myeloperoxidase (MPO) are reportedly implicated in IR induced liver injury^8^. In particular, MPO is highly expressed in neutrophils and serves as a marker of neutrophil accumulation. MPO contributes to oxidative stress in the tissue by reacting with hydrogen peroxide^8^. MPO activity was measured in the ischemic liver lysate (Fig. 5a). 12-mer-1 treatment decreased MPO activity 60% compared to the IR group. In contrast, MPO activity was nearly identical between the 12-mer-3 and IR groups (96.00 vs 96.33 U/g protein, respectively). Furthermore, neutrophil accumulation was measured in the ischemic tissue by immunohistochemistry (Fig. 5b–f). Similar to the MPO trend, 12-mer-1 decreased neutrophil infiltration whereas 12-mer-3 did not (12-mer-1 vs IR *P* = 0.0142, 12-mer-3 vs IR *P* = 0.0705).

### Anticoagulation alone is not sufficient for hepatoprotection

**Figure 6:**
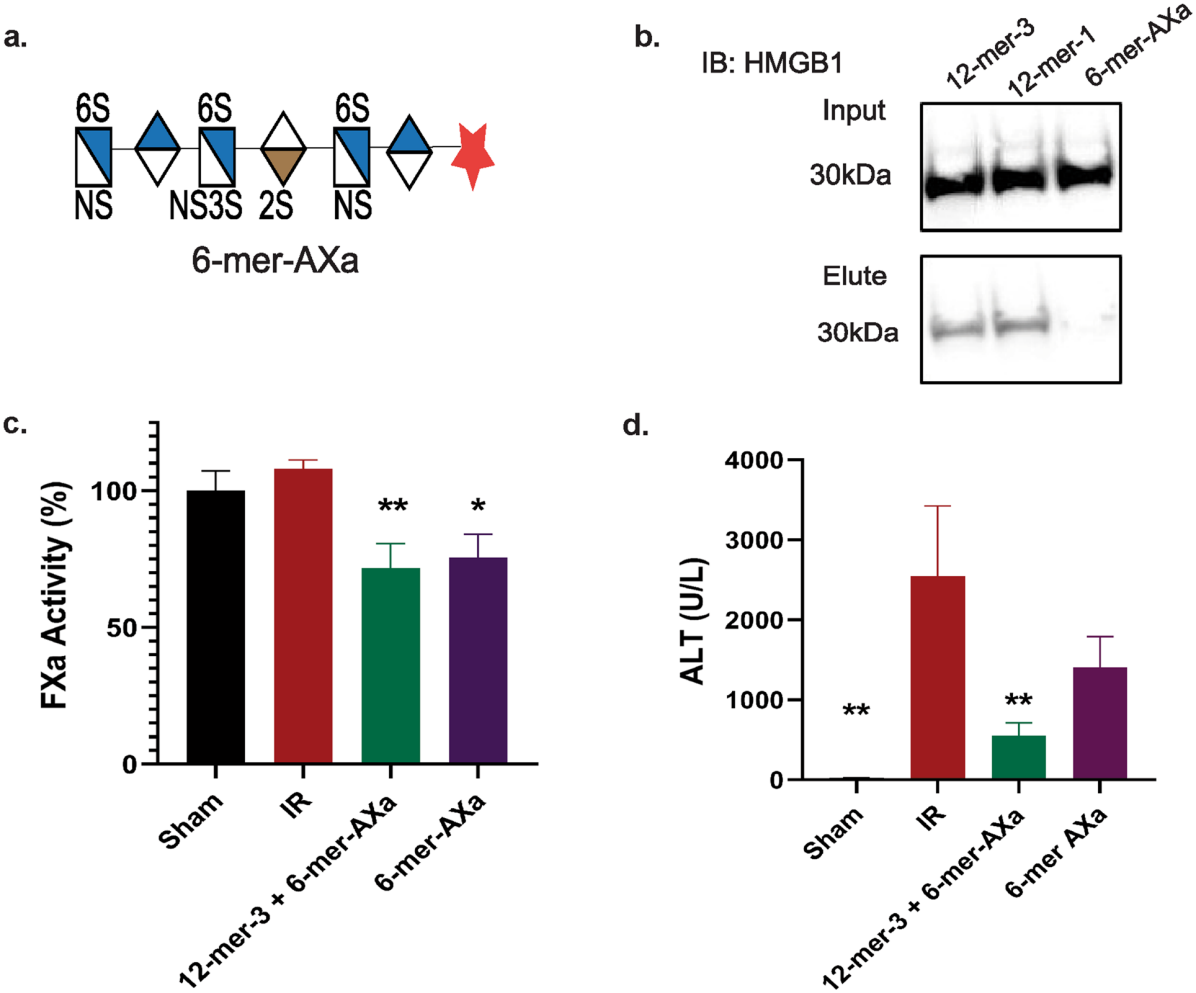
HMGB1 binding and anticoagulation are necessary for hepatoprotection. (**a**) Illustration of 6-mer-AXa structure. (**b**) Western analysis of HMGB1 pulldown from liver lysate using biotinylated 6-mer-AXa with 12-mer-3 and 12-mer-1 as a positive control. (**c**) Plasma FXa activity was measured from mice that underwent a sham or IR procedure with administration of 12-mer-3 + 6-mer-AXa or 6-mer-AXa alone. IR vs. 12-mer-3 + 6-mer-AXa, *P* = 0.0089; IR vs 6-mer-AXa, *P* = 0.0252. (**d**) Plasma ALT. IR vs Sham, *P* = 0.0026; IR vs 12-mer-3 + 6-mer-AXa, *P* = 0.0086; IR vs 6-mer-AXa, *P* = 0.1440. Data represent mean ± SEM. Sham n = 4, IR n = 3–5, 12-mer-3 + 6-mer-AXa n = 6, 6-mer-AXa n = 6.

Next, we investigated if hepatoprotection requires anticoagulant activity. To accomplish this, we used a 6-mer-AXa oligosaccharide (Fig. 6a). Similar to 12-mer-1, 6-mer-AXa has anticoagulant activity through inhibition of FXa as previously demonstrated^15^. Biotinylated 6-mer-AXa does not pull down HMGB1 from liver lysate (Fig. 6b), and thus serves as a control for anticoagulant activity without HMGB1 binding. To determine if HMGB1 binding and anticoagulation from a HS oligosaccharide are both necessary for hepatoprotection after IR, we used a 6-mer-AXa oligosaccharide alone or in combination with 12-mer-3. Anticoagulant levels are similar between both treatment groups (Fig. 6c), however only the combination treatment of oligosaccharides having HMGB1 binding ability (12-mer-3) and anticoagulant activity (6-mer-AXa) decrease plasma ALT after IR with statistical significance. There was no statistical difference in the concentration of ALT between IR-injured group and 6-mer AXa-treated group (6-mer-AXa vs. IR, *P* = 0.1440). This result demonstrates that both activities, either stemming from one compound with dual activity or a combination of two compounds with separate functions, is necessary for hepatoprotection.

## Discussion

We recently reported the beneficial effect of HS oligosaccharides in acetaminophen-induced liver injury and ascribed the protective mechanism to inhibiting HMGB1 mediated neutrophil infiltration to the liver^20^. HMBG1 has also been implicated in sterile inflammation after liver IR^6,7,17^. Liver IR and acetaminophen-induced liver injury are alike in that both are acute liver injuries that involve neutrophil infiltration and sterile inflammation. During liver IR, neutrophils are rapidly recruited during the reperfusion phase to the post-ischemic tissue^8^. After neutrophils migrate into the liver, they release cytotoxic compounds, including reactive oxygen species and proteases, to clear damaged tissues^21^. Neutrophils and their potent cargo are key effectors in sterile inflammation due to the lack of specificity for damage vs healthy tissue. As a result, neutrophil recruitment continues and perpetuates inflammation.

Unlike acetaminophen-induced liver injury, liver IR is reported to involve coagulation disturbances in addition to inflammation and thus described as thromboinflammation^3^. Anticoagulant HS oligosaccharides were ineffective in acetaminophen-induced liver injury^20^; however, since liver IR involves thromboinflammation we included anticoagulant 12-mer-1 in our study to explore how anticoagulant activity and HMGB1 binding effected hepatoprotection in liver IR injury. In doing so, we are demonstrating that synthetic HS oligosaccharides are a potential therapeutic in another disease model and its protection role is not solely limited to acetaminophen-induced liver injury.

In the present work, we explore the structure–activity relationship of HS oligosaccharides for HMGB1 binding by screening a panel of 12-mer oligosaccharides with various sulfation patterns. 12-mer-1 and 12-mer-3, which are both highly sulfated oligosaccharides, were the only successful compounds to pull down HMGB1 from liver lysates. However, in vivo we observed that 12-mer-1 but not 12-mer-3 decreased ALT and necrosis in the ischemic liver lobe. 12-mer-1’s anti-inflammatory activity is associated with the ability to bind to HMGB1, decrease tissue MPO, and decrease neutrophil accumulation in ischemic liver lobe. Interestingly, binding to HMGB1 is not sufficient for hepatoprotection as demonstrated by 12-mer-3 in vivo. We show that both anticoagulation and anti-inflammatory activity are necessary to achieve hepatoprotection by using 6-mer-AXa in combination with 12-mer-3 or alone in the IR model. In this way, anticoagulation in the absence or presence of HMGB1 binding was examined. Treatment with 6-mer-AXa alone did not decrease ALT that has statistical significance, however the combination treatment did. Similar to previously published work, a selective small molecule FXa inhibitor DX9065a had no hepatoprotection in a rat liver IR model^22^. We attribute 12-mer-1’s hepatoprotective effect to the dual activities of anticoagulation and anti-inflammation as both mechanisms are essential in the pathophysiology of liver IR.

In our previous work, 12-mer-1 was successfully investigated as an anticoagulant treatment in a model of venous thrombosis induced by stenosis^16^. In this work, we build on 12-mer-1’s profile by demonstrating that it is an active anti-inflammatory agent as well as an anticoagulant. In addition to 12-mer-1’s protective mechanism in liver IR, 12-mer-1 also has several favorable drug-like properties. For future studies, we will consider the renal clearance of 12-mer-1 since most liver transplant patients also have impaired renal function^23^. The renal clearance impairment of 12-mer-1 was demonstrated using a kidney IR model^16^. However, 12-mer-1 is amenable to dose adjustments since it is a homogeneous compound with uniform anticoagulant activity and potentially a safe option for renally impaired patients. Bleeding issues have been reported, with 9% of liver transplant recipients on heparin therapy requiring surgical intervention for bleeding complications^24^. LMWH lowers the bleeding risk but it is incompletely reversed by protamine^16^. 12-mer-1 anticoagulant activity is reversible by protamine which adds an additional benefit to ameliorate bleeding complications^16^. Thus, controlling the dose and having the potential of reversibility by protamine are important aspects of 12-mer-1. Additionally, 12-mer-1 displayed no toxicity in a rat model at elevated doses^16^. Therefore, the anti-thromboinflammatory properties, reversibility by protamine, lack of toxicity and ability to precisely control the dose make 12-mer-1 an appealing therapeutic for liver transplant/IR patients. Future development of 12-mer-1’s drug-like profile will require pharmacokinetic studies to investigate the relationship of 12-mer-1 dose and response against liver IR injury.

The chemoenzymatic synthesis technology generates structurally defined HS oligosaccharides. As heparin is becoming increasingly recognized for its anti-inflammatory properties^25^, our technology will help to transform the therapeutic field from one using heterogeneous mixtures of oligosaccharides to a new class of homogeneous, precision-based oligosaccharide therapeutics.

## Materials and methods

### Chemoenzymatic synthesis of oligosaccharides

The synthesis of the 12-mers and 6-mer-AXa has been previously described^15^. Briefly for the synthesis of 12-mer-4, glucuronic acid-pNP was elongated with UDP-GlcNTFA (step **a**) and UDP-GlcA (step **b**) using pmHS2 to reach 12-mer. Next, GlcNTFA was deprotected using LiOH then *N-*sulfated with NST (step **c**) to yield 12-mer *N*-sulfo glucosamine residues. Next, 6-O-sulfation was installed using 6-OST-1 and 6-OST3- (step **d**) to yield 12-mer-4 GlcNS6S-GlcA-GlcNS6S-GlcA-GlcNS6S-GlcA-GlcNS6S-GlcA-GlcNS6S-GlcA-GlcNS6S-GlcA-pNP. 12-mer-1, -2, and -3 were synthesized by elongating the monosaccharide to a pentasaccharide intermediate. Next, GlcNTFA was deprotected using LiOH then *N-*sulfated with NST to yield 5-mer with two *N*-sulfo glucosamine residues. 5-mer NS was elongated with UDP-GlcNTFA to yield a 6-mer intermediate. The glucuronic acid residue in between the two *N-*sulfo glucosamine residues undergoes epimerization by C5-epimerase and *2-*O-sulfation by 2-OST (step **e**) to yield the 6-mer intermediate GlcNTFA-GlcA-GlcNS-IdoA2S-GlcNS-GlcA-pNP. To generate 6-mer-AXa, this 6-mer intermediate underwent step **c, d, e,** and 3*-O*-sulfation by 3-OST1 (step **f**) to give GlcNS6S-GlcA-GlcNS6S3S-IdoA2S-GlcNS6S-GlcA-pNP. To generate 12-mer-2, the 6-mer intermediate underwent steps **b, c, a,** and **e** repeated three times to yield GlcNS-GlcA-GlcNS-IdoA2S-GlcNS-IdoA2S-GlcNS-IdoA2S-GlcNS-IdoA2S-GlcNS-GlcA-pNP. 12-mer-2 was converted to 12-mer-3 by 6*-O*-sulfation of GlcNS residues using 6-OST-1 and 6-OST-3 (step **e**) to give the structure GlcNS6S-GlcA-GlcNS6S-IdoA2S-GlcNS6S-IdoA2S-GlcNS6S-IdoA2S-GlcNS6S-IdoA2S-GlcNS6S-GlcA-pNP. Lastly, 12-mer-3 was converted to compound 12-mer-1 by step **f** to give the structure GlcNS6S-GlcA-GlcNS6S3S-IdoA2S-GlcNS6S-IdoA2S-GlcNS6S-IdoA2S-GlcNS6S-IdoA2S-GlcNS6S-GlcA-pNP. The purity of different 12-mers and 6-mer-AXa were > 95% as measured by high resolution DEAE-HPLC. The chemical structures were confirmed by electrospray ionization mass spectrometry (ESI–MS) and NMR^15^.

The oligosaccharides were converted to biotinylated versions as previously described^20^. Briefly, 12-mers and 6-mer-AXa with a pNP tag (5–10 mg) and 0.5 mg Pd/C were dissolved in 20 mM NaOAc, pH 5.0 in a total volume of 4 ml. Reaction mixture was vacuumed and refilled with H_2_ three times. The reaction was then incubated at room temperature for 4 h. After that, it was filtered to remove charcoal. The filtered solution was adjusted to pH 8.5 using 500 mM Na_2_HPO_4_. Succinimidyl 6-azidohexanoate (20 molar equivalent of starting oligosaccharides) was added and incubated at 37 °C overnight. Reaction was purified by DEAE-HPLC column to generate azido tagged oligosaccharides. PBS (pH 7.4) buffer was bubbled with N_2_ for 5 min to prepare the sample solution of 0.1 M CuSO_4_, 0.1 M Tris(3-hydroxypropyl-triazolylmethyl)amine (THPTA) (Sigma), 0.15 M sodium ascorbate, 0.01 M azido tagged oligosaccharides and 0.02 M biotin-PEG_4_-alkyne (Sigma). The mixture of 400 µl THPTA and 80 µl CuSO_4_ was vortexed, then 160 µl sodium ascorbate, 200 µl azido tagged oligomers and 200 µl biotin-PEG4-alkyne was added and bubbled with N_2_ for 2 min, then incubated at 37 °C overnight. The reaction was purified by DEAE-HPLC column to generate biotinylated products. The biotinylated 12-mers and 6-mer-AXa products were confirmed by ESI–MS.

### Affinity purification of HMGB1 from liver lysate

6-mer-AXa and 12-mer biotinylated oligosaccharides were using to affinity purify HMGB1 from liver lysate following a previously described method^20^. Briefly, liver lysate was prepared by snap freezing tissue in liquid nitrogen at the time of sacrifice. The tissue was mechanically homogenized in buffer containing 200 mM MES, 500 mM phosphate, and 1 mM EDTA at pH 6 followed by three rounds of freeze thawing. The lysed sample were centrifuged at 10,000×*g* for 15 min at 4 ºC. Biotinylated HS oligosaccharides (final concentration 0.1 mM) were mixed with 20 μl of fresh liver lysate (~ 0.6 mg) in 100 mM NaCl 20 mM HEPES pH 7.2 and incubated overnight at 4 °C. The purification and biotinylated HS bound complex where achieved using avidin-Sepharose and increasing concentration of NaCl washes. The elution of each sample was separated by gel electrophoresis, transferred to nitrocellulose membrane, and blotted for HMGB1 using anti-HMGB1 primary antibody (Abcam) followed by anti-rabbit HRP (Abcam).

### Determination of the in vitro and ex vivo anti-FXa activity of oligosaccharides

Assays were based on a previously published method. Briefly, human FXa (Enzyme Research Laboratories) was diluted to 50 U ml^−1^ with PBS. The chromogenic substrate S-2765 (Diapharma) was diluted to 1 mg ml^−1^ in water. For in vitro studies, fondaparinux and 12-mer oligosaccharides were dissolved in PBS at various concentrations (0–131 nM). 16 μl of sample was incubated with 60 μl of 35 μl ml^−1^ antithrombin (Cutter Biologics) for 2 min at room temperature. Next, 100 μl of FXa was added and incubated for 4 min at room temperature. 30 μl of S-2765 substrate was added and the absorbance of the reaction mixture was measured at 405 nm continuously for 5 min. PBS serves as a control sample. The maximum slope for each sample was convert to percent FXa activity by dividing by the maximum slope for the control sample.

For ex vivo studies, mouse plasma collected after the 6 h reperfusion period and assayed the same as described above.

### Liver ischemia–reperfusion surgery design

Liver ischemia–reperfusion (IR) surgery was performed by the Animal Surgery Core Laboratory of the McAllister Heart Institute, University of North Carolina Chapel Hill, NC. The mouse experiments were approved by the UNC Animal Care and Use Committees and complied with National Institutes of Health guidelines. Male C57BL/6J mice, approximately 8 weeks old, were used in the IR surgeries. Mice received a subcutaneous (SC) injection of 1 mg/kg oligosaccharide or the equivalent volume of saline 30 min prior to the surgical procedure. For the combination treatment of 12-mer-3 + 6-mer-AXa, equal concentration of each oligosaccharide was combined into a single solution. Under ketamine/xylazine anesthesia, an abdominal midline incision was made to expose the portal vein. A clamp was placed on the portal vein and bile duct to three major liver lobes to cause a 70% hepatic ischemia. Visible blanching of the ischemic liver lobes confirmed correct placement of the clamp. A temporary stitch closure of the muscle and skin over the clamp was used to prevent dehydration during the ischemia phase. Mice stayed on a heating pad and under anesthesia during the ischemia phase (60 min). The clamp was removed after 60 min and the ischemia liver lobes regained their red color as blood began to reperfusion the tissue, an indicator of correct reperfusion occurring. The incisions were closed in two layers with 5–0 silk sutures and the mice returned to their active state (no anesthesia used during the reperfusion phase). After 6 h, the mice were re-anaesthetized, blood was drawn via cardiac puncture and ischemia liver lobes were harvested for histology (fixed in 10% formalin).

### Evaluation of liver I/R injury

Plasma ALT was measured using the ALT Infinity reagent (Thermo Fisher) following the manufacturer’s instructions.

### Histology/immunohistochemistry

Ischemia liver tissues were fixed in 10% neutral buffered formalin for 24 h at room temperature, paraffin-embedded, and sectioned. Liver sections (4 μm) were stained with hematoxylin–eosin (H&E) or immunostained with monoclonal antibodies anti-neutrophil (Abcam, Ab 2557, NIMP-R14) followed by goat anti-rat or goat anti-rabbit biotinylated secondary antibodies (Abcam). Embedding, sectioning and H&E staining were performed at the Animal Histopathology and Laboratory Medicine Core Facility at UNC Chapel Hill. H&E analyses were performed by the Translational Pathology Laboratory Core Facility at UNC Chapel Hill using Aperio ImageScope Software (Leica Biosystems, Concord, Canada). IHC images were captured using an HD camera attached to a bright field microscope (Leica DM 1000 LED, Leica Microsystems Inc., IL, USA) and were processed using ImageJ. For neutrophil quantitation, five 100 × images were randomly selected for each sample and the average neutrophils/field were reported.

### MPO activity

Ischemia liver lobe was mechanically homogenized in 50 mM CTAB 50 mM potassium phosphate pH 6 at a ratio of 100 μl buffer per 10 mg tissue. Samples were centrifuged at 15,000×*g* for 20 min 4 °C and the supernatant was collected and stored at − 20 °C. Total protein concentration was measured by Bradford assay. 10 μl of liver lysate was incubated with 80 μl of 0.75 mM H_2_O_2_ and 110 μl of TMB (TMB liquid reagent, ready-to-use, Sigma) for 10 min at 37 °C with gentle agitation. The reaction was stopped by addition of 2.5 M H_2_SO_4_ and read at 450 nm. Activity (U/g protein) was calculated as absorbance of sample minus the absorbance of the blank divided by incubation time. This value was normalized by the protein concentration.

### Statistical analysis

All data are expressed as mean ± SEM. Statistical significance between experimental and control groups were analyzed by two-tailed unpaired Student *t* test and between multiple groups by one-way ANOVA followed by Dunnett’s multiple comparison’s test using GraphPad Prism software (version 7.03; GraphPad Software, Inc., https://www.graphpad.com/scientific-software/prism/).

## Supplementary information

Supplementary Information.
