# Supplementary material for: Synthetic anticoagulant heparan sulfate attenuates liver ischemia reperfusion injury
Source: Sci Rep. 2020 Oct 14;10:17187. doi: 10.1038/s41598-020-74275-7 (PMC7566620; doi:10.1038/s41598-020-74275-7)
Supplement: Supplementary file 1 — Supplementary Information. [file 41598_2020_74275_MOESM1_ESM.docx]

**SYNTHETIC ANTICOAGULANT HEPARAN SULFATE ATTENUATES LIVER ISCHEMIA REPERFUSION INJURY**

**Supplementary Information**

**Katelyn Arnold^1^, Yongmei Xu^1^, Yi-En Liao^1^, Brian C. Cooley^2^, Rafal Pawlinski^3^ and Jian Liu*^1^**

1. Division of Chemical Biology and Medicinal Chemistry, Eshelman School of Pharmacy, University of North Carolina, Chapel Hill, NC, USA.
2. Department of Pathology and Laboratory Medicine, University of North Carolina, Chapel Hill, NC, USA
3. UNC Blood Research Center, Division of Hematology/Oncology, Department of Medicine, University of North Carolina, Chapel Hill, NC, USA.

*Correspondence: [jian_liu@unc.edu](mailto:jian_liu@unc.edu)

8

7

6

5


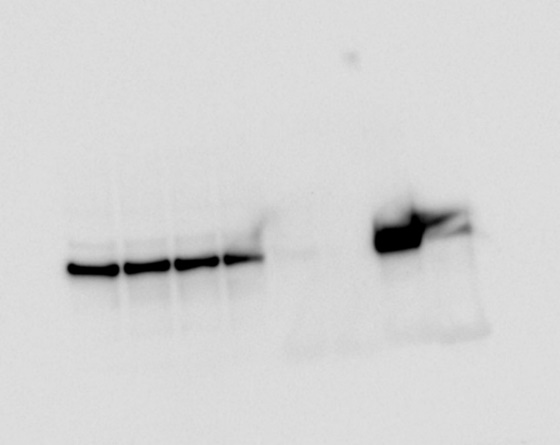


A.

30kDa


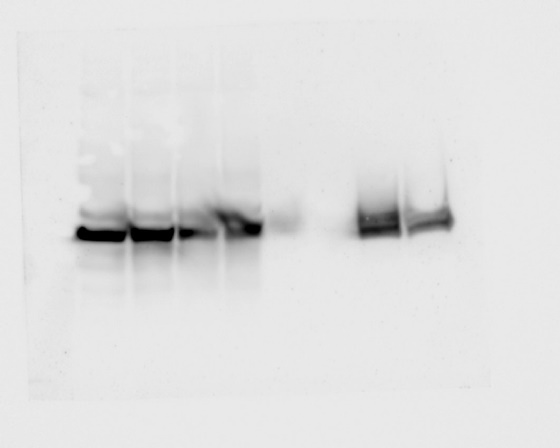


B.

30kDa

4

3

2

1

Supplementary Fig 1. Full western blot images from HMGB1 pulldown using oligosaccharides. **A.** Sample input. Lane 1: 12-mer-4; Lane 2: 12-mer-2; Lane 3: 12-mer-3; Lane 4: 12-mer-1. The rest of the blot is from another experiment and is not relevant to this result. **B.** Sample elution. Lane 5: 12-mer-4; Lane 6: 12-mer-2; Lane 7: 12-mer-3; Lane 8: 12-mer-1. The rest of the blot is from another experiment and is not relevant to this result.


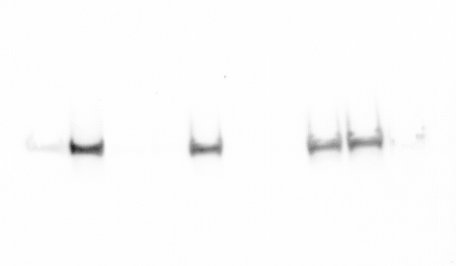


4

5

B.


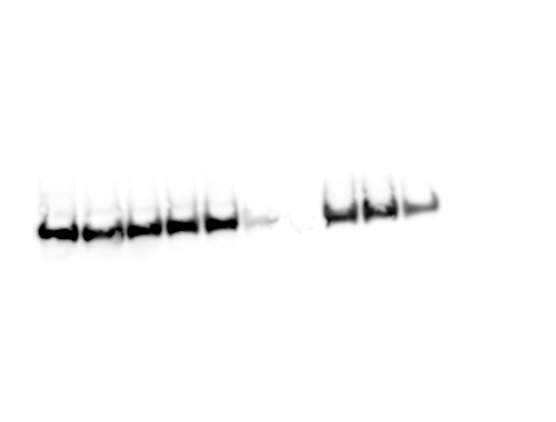


1

2

A.

30kDa

6

3

Supplementary Fig 2. Full western blot images from HMGB1 pulldown using 12-mer-1 and 6-mer-AXa oligosaccharides. **A.** Sample input. Lane 1: 12-mer-3; Lane 2: 12-mer-1; Lane 3: 6-mer-AXa. The rest of the blot is from another experiment and is not relevant to this result. **B.** Sample elution. Lane 4: 12-mer-3; Lane 5: 12-mer-1; Lane 6: 6-mer-AXa. The rest of the blot is from another experiment and is not relevant to this result.
